# MiR-145-5p modulates collagen production and fibroblast behaviour after cardiac injury partially via COL5A1

**DOI:** 10.1007/s00395-026-01199-0

**Published:** 2026-08-03

**Authors:** Hong Wang, Jere Paavola, Sanni Perttunen, Katariina Immonen, Ian Hägerström, Suneeta Narumanchi, Karri Kalervo, Johanna Tolva, Juha Sinisalo, Mikko I. Mäyränpää, Heikki Ruskoaho, Riikka Kosonen, Vesa M. Olkkonen, Mika Laine, Ilkka Tikkanen, Päivi Lakkisto

**Affiliations:** 1 Minerva Foundation Institute for Medical Research, Biomedicum Helsinki 2U, Tukholmankatu 8, 00290 Helsinki, Finland; 2 Heart and Lung Centre, University of Helsinki and Helsinki University Hospital, 00014 Helsinki, Finland; 3 Abdominal Centre Gastrointestinal Surgery, University of Helsinki and Helsinki University Hospital, 00014 Helsinki, Finland; 4 Department of Pathology, University of Helsinki and Helsinki University Hospital, 00014 Helsinki, Finland; 5 Drug Research Program, Division of Pharmacology and Pharmacotherapy, Faculty of Pharmacy, University of Helsinki, 00014 Helsinki, Finland; 6 Department of Anatomy Faculty of Medicine, University of Helsinki, 00014 Helsinki, Finland; 7 Abdominal Centre Nephrology, University of Helsinki and Helsinki University Hospital, 00014 Helsinki, Finland; 8 Department of Clinical Chemistry, University of Helsinki and Helsinki University Hospital, 00014 Helsinki, Finland

**Keywords:** Cardiac fibrosis, Cardiac scar resolution, MiR-145-5p, COL5A1

## Abstract

In contrast to humans, zebrafish hearts after cryoinjury undergo transient fibrotic scarring that subsequently resolves and renews with functional cardiomyocytes. To understand the molecular mechanisms underlying cardiac scarring and fibrotic resolution in zebrafish, we investigated cardiac transcriptomic responses in zebrafish at 14 days post-cryoinjury. Principal component analyses revealed distinct transcriptome clusters corresponding to healthy and injured hearts, highlighting significant changes during cardiac regeneration. Gene set enrichment analyses indicated that extracellular matrix organization and inflammatory response pathways were activated in cryoinjured hearts, while mitochondrial organization and oxidative phosphorylation pathways were downregulated. Notably, miR-145-5p was downregulated and *col5a1* upregulated in the cryoinjured hearts, contrasting with the expression patterns of miR-145-5p and COL5α1 in the left ventricles of ischemic cardiomyopathy patients. Prediction of miRNA-mRNA interaction and a dual luciferase assay identified *COL5A1* as a target of miR-145-5p. Inhibition of miR-145-5p in human cardiac fibroblasts increased COL5A1 and α-SMA, decreased COL1A, and stimulated fibroblast differentiation, proliferation, and migration. Conversely, overexpression of miR-145-5p led to the opposite effects. Downregulation of COL5A1 suppressed fibroblast proliferation and migration, which could be rescued by inhibition of miR-145-5p. Moreover, downregulation of *col5a1* attenuated cardiac fibrotic scar resolution in zebrafish. Interestingly, downregulation of COL5A1 or inhibition of miR-145-5p diminished the expression of integrin subunits ITGβ3 and ITGβ5. Furthermore, inhibition of miR-145-5p potentiated the suppression of TGF-β/SMAD signalling and the enhancement of fibroblast proliferation mediated by inhibiting integrin αvβ3 and αvβ5 with cilengitide. Collectively, miR-145-5p modulates collagen production and fibroblast behaviour partially via targeting COL5A1 and through integrin-mediated pathways.

**Supplementary Information:**

The online version contains supplementary material available at https://doi.org/10.1007/s00395-026-01199-0.

## Introduction

Myocardial infarction (MI) evokes adverse remodelling, leading to cardiomyocyte loss, fibrotic scar formation, cardiac dysfunction, and progressively heart failure in human [21]. This is because of persistent fibrosis and the limited capacity of cardiomyocyte renewal in adult hearts [21]. By contrast, zebrafish possess a unique ability of cardiac tissue regeneration following injury throughout their lives [39]. Zebrafish cardiac regeneration requires early macrophage infiltration, rapid revascularization, transient fibrosis followed by resolution and cardiomyocyte proliferation [20, 25]. Scar formation is crucial to prevent ventricular rupture, however, the initial fibrotic tissues are resolved and replaced with functional cardiomyocytes. The efficiency of resolving fibrotic tissue is an important factor in determining cardiac regenerative outcome [19].

Cardiac fibroblasts play an essential role in cardiac fibrosis and scar formation. Following MI, fibroblasts exhibit pronounced plasticity and undergo rapid phenotypic transitions from quiescence to a pro-inflammatory and matrix-degrading phenotype, then to a matrix-synthetic, proliferative myofibroblast phenotype, and revert to quiescence as the scar matures [24]. Activated myofibroblasts are the main matrix-synthetic cells in the infarcted heart and produce matricellular macromolecules and structural extracellular matrix proteins, of which type I (COL1) and type III collagen (COL3) account for approximately 90–95% of the cardiac collagens [15], whereas type V (COL5) is minimally expressed in the heart and a minor component of scar tissue [57]. During scar maturation, fibroblasts exhibit disassembly of alpha smooth muscle actin (αSMA)-decorated stress fibers and produce matrix-crosslinking enzymes to form a stable collagen-based scar. In zebrafish hearts after cryoinjury, collagen-rich scars typically mature at approximately 14–21 days post-injury (dpi) and then progressively regress as the myocardium regenerates [17, 20]. The levels of collagens and fibrillin show a statistically significant decrease, while collagen degradation mediated by certain types of matrix metalloproteinases increases, leading eventually to complete collagen disappearance [17]. The functional diversity of fibroblasts may reflect sequential activation of distinct fibroblast subpopulations or result from coordinated responses of the fibroblasts to the dynamic changes in their microenvironment. The molecular basis for the phenotypic transitions of cardiac fibroblasts in the phases of infarct healing remains poorly understood. 

A growing body of evidence demonstrates that microRNAs (miRNAs) that induce translational repression and mRNA degradation play crucial roles in regulating the activation of cardiac fibroblasts following cardiac injury [3, 12, 41, 58]. miRNAs can either promote or inhibit the formation and resolution of cardiac scar tissue, thereby influencing cardiac size and overall function after MI [3, 41, 58]. Comparative transcriptome profiles of injured and healthy zebrafish hearts identified miRNAs as regulators of heart regeneration [27], highlighting their potential in targeted therapy after myocardial infarction. Here, we monitored the transcriptomic response after 14 days cryoinjury to decipher the potential miRNAs regulating zebrafish scar remodelling during cardiac regeneration.

## Methods and materials

### Zebrafish husbandry and ventricular cryoinjury

Zebrafish were obtained from a breeding line maintained in the zebrafish core facility at the University of Helsinki. They were housed at 28 °C with a 14:10 h light:dark cycle. Ventricular cryoinjury was conducted as described previously [36]. Briefly, zebrafish were anesthetized with 0.03% tricaine and a small incision was made on the ventral side of the fish for direct access to the heart. A metal probe precooled in liquid nitrogen was placed on the ventricular surface for 15 seconds. The sham-operated zebrafish underwent the same procedures using a metal probe kept at room temperature. After two weeks post-injury, the hearts were dissected, snap-frozen in liquid nitrogen, and stored at −80°C. Given that the surgical impact appears to be the greatest at one dpi, but negligible at later time points [27], we thus employed healthy hearts as controls.

To assess the role of Col5A1 in fibrotic scar remodelling, thirty-two adult zebrafish were randomly assigned to two groups matched by body size: a negative control (NC) group and a COL5A1 GapmeR G2 (COL5A1 G2) group. After anaesthesia, fish received a single intraperitoneal injection of either antisense LNA™ COL5A1 HS DR2 GapmeR (ID 339523 LG00851814-FDA, A*G*T*A*T*T*C*A*C*C*A*T*C*T*G*G, in vivo ready, Qiagen) or antisense LNA™ Negative Control A (ID 339515 LG00000002-FDA, A*A*C*A*C*G*T*C*T*A*T*A*C*G*C, in vivo ready, Qiagen) at a dose of 2.5 mg/kg body weight, followed by ventricular cryoinjury. The GapmeRs were designed to target *col5a1* with 100% sequence homology to the human *COL5A1*. Eleven fish survived the procedure in the NC group and twelve in COL5A1 G2 group. Cardiac function was monitored at 0 (baseline), 7, and 14 days post-injury (dpi). Hearts, livers, and kidneys were then collected. Six hearts were fixed in 10% formalin and embedded in paraffin. Organs from two fish were pooled, snap frozen on dry ice, and stored at −80°C, except for one NC sample, which was processed individually.

### Human cardiomyopathy patients

The left ventricular (LV) samples of ischemic cardiomyopathy (ICM) patients were obtained from patients undergoing cardiac transplantation in Helsinki University Hospital between 2014 and 2019. Control samples were derived from the LVs of organ donors without cardiac disease, whose hearts could not be used as whole organ grafts due to tissue type or size mismatch. The myocardium samples were taken from the non-infarcted regions and immediately frozen in isopentane solution (2-methylbutane) cooled in liquid nitrogen and stored at −80°C. The age, gender, and medical and medication history of the patients have been previously described [50, 51].

### Echocardiography of adult zebrafish

Echocardiography of adult zebrafish was performed with Vevo 2100^®^ Image System and Vevo Imaging Station (VisualSonics, Amsterdam, Netherlands) equipped with a high frequency transducer (MS700, 30–70MHz) as described previously [53]. In brief, fish were anaesthetized with 0.02% tricaine in system water. Echocardiographic images were acquired in longitudinal axis (LAX) view to record B-mode videos. After echocardiography, fish were placed into a tank filled with fresh system water and usually recovered within 20 seconds.

Image analysis was conducted off-line with the Vevo Lab™ analysis software (VisualSonics) by experienced personnel. Briefly, speckle-tracking analysis of ventricular wall motion was performed to assess ejection fraction (EF) and global longitudinal strain (GLS) from five consecutive cardiac cycles. Ventricular inner border of compact myocardium was defined as the internal chamber. Subsequently, the software calculated EF, strain, and heart rate (HR). EF is defined as the difference between end-diastolic volume (EDV) and end-systolic volume (ESV) divided by EDV, EF = (EDV–ESV)/EDV. Strain (ε) is defined as the change in the length (L) of a deformed object divided by the original length (L_0_), ε = L–L_0_/L_0_. GLS represents the average of six segmental strain values of myocardial wall.

### RNA sequencing

RNA from snap-frozen zebrafish hearts (MI *n* = 4, Ctrl *n* = 4) was extracted with TRIsure™ Kit (Bioline Ltd, United Kingdom) following the manufacturer’s instructions. Briefly, heart was homogenized with a Tissue-Tearor (BioSpec, Bartlesville, OK, USA) in 0.8 mL of TRIsure. After incubating for 5 minutes at room temperature (RT), 250 μL of chloroform was added and mixed vigorously for 15 seconds. Aqueous phase was separated by centrifuge at 12,000 × g for 15 minutes at 4 °C and transferred to a fresh tube for RNA extraction. To precipitate RNA, 600 μL of cold isopropyl alcohol was added. Followed incubation for 10 minutes at RT, RNA was precipitated with centrifuge at 12,000 × g for 10 minutes at 4°C. Pellet was washed with 800 μL of 75% ethanol, air-dried, and dissolved in DEPC-treated water.

High-throughput RNA-sequencing was performed with Illumina HiSeq™ 2000 (Illumina, San Diego, CA, USA) at BGI (Shenzhen, China) as pair-end sequencing for read of 100 bp. The data quality was analysed with FastQC (http://www.bioinformatics.babraham.ac.uk/projects/fastqc/) and summarized with MultiQC [13]. A light quality trimming was applied to the data with the Trimmomatic software [6]. The sample reads were aligned against Danio rerio genome GRCz11(GCA_000002035.4) (https://www.ncbi.nlm.nih.gov/assembly/GCF_000002035.6/) or miRBase release 22 [28] with STAR-aligner [9]. Alignment statistics were collected with the Qualimap tool [37]. Read quantifications were calculated with FeatureCounts software [30]. Read qualities were accessed automatically in the normalization and statistical processes inside DESeq2 [32].

### Analysis of differentially expressed genes and miRNAs

The differential expression analyses were performed with the DESeq2 software in R environment [32]. Negative binomial linear model and Wald test were used to produce *p*-values. All *p* values were adjusted for multiple comparisons using Benjamini-Hochberg correction. Probe sets with an adj. *p-*value < 0.05 were considered significantly differentially expressed. The basic gene annotations came from Ensembl Release 92 [56] and the required data quires were done in R environment with BioMart tool [11] (Supplementary Table 1). In terms of miRNA analysis, infarct 3 appeared to be an outlier and was thus excluded (Supplementary Table 2). Omitting this one sample did not change the most top differentially expressed miRNAs.

### Gene set enrichment analysis

Gene set enrichment analysis (GSEA) was performed with the GSEA program (https://www.gsea-msigdb.org/gsea/index.jsp) using all genes to calculate enrichment scores. The enrichment scores were normalized to account for the size of the translational gene set. Genes were ranked according to the correlation between their expression values and the phenotype class distinction using the signal-to-noise ratio. The statistical significance of the normalized enrichment score was estimated using gene set-based permutation test [45].

Similar enrichment analysis was conducted with the general enrichment tool in R (GAGE) using fold changes. The result was subjected to pathway analysis against Kyoto Encyclopedia of Genes and Genomes (KEGG) (https://www.genome.jp/kegg/pathway.html) and Gene Ontology (GO). The enriched pathways were visualized with Pathview tool (https://bioconductor.org/packages/release/bioc/html/pathview.html).

### Collagen network analysis

The protein-protein interaction network of all COL genes detected in the RNA-seq dataset from hearts of ICM patients (Supplementary Table 3) was constructed using the STRING database (https://string-db.org/) with a confidence score threshold of 0.7. The resulting COL network was partitioned into five clusters using K-means clustering. Relative expression across cardiac cell types was extracted from summarized single-cell RNA-sequencing profiles provided by the Human Protein Atlas (https://www.proteinatlas.org/). Expression values were Z-score standardized prior to heatmap generation, which was performed in R (version 4.3.1).

### Prediction of miRNA-mRNA interactions

Predicted targets for the differentially expressed miRNAs (adj. *p*-value < 0.05) between healthy and cryoinjured hearts were obtained from TargetScan 6.2 (https://www.targetscan.org/vert_61/). Pearson correlations and corresponding *p*-values were calculated between mRNA and miRNA normalized expression using all healthy and cryoinjured heart samples. Correlated mRNA-miRNA pairs were extracted using a Pearson correlation* p*-value <0.05. The predicted targets that were assigned a total context + score in the top 25 percentile and significantly differentially expressed (adj. *p*-value <0.05) between healthy and cryoinjured hearts were selected. The interactions between miRNAs and their target genes were drawn with RAWGraphs (https://www.rawgraphs.io/).

### RNA extraction and real-time quantitative RT-PCR

Owing to the conservation of miRNA gene families among species, we employed human miRNA primers to analyse homologous miRNA genes in zebrafish. Sequence search against miRBase database (http://www.mirbase.org) using the primers as queries identified the respective homologous miRNAs in zebrafish. To analyse miRNA and gene expressions, 30 zebrafish were randomly divided into three groups: control, sham, and cryoinjury. RNA was extracted from 3–4 pooled zebrafish hearts with miRNeasy Mini Kit (Qiagen, Hilden, Germany) according to the manufacturer’s instructions. Reverse transcription was performed with a miScript II RT kit (Qiagen). Quantitative PCR was performed with a miScript primer assay (Qiagen) and miScript SYBR Green PCR kit (Qiagen) using Light Cycler 480 II instrument (Roche Applied Science, Penzberg, Germany). Small Nucleolar RNA, C/D Box 61 (SNORD 61, Catalog #MS00033705, Qiagen) served as an internal control for normalization. The expression levels of investigated miRNAs in sham-operated hearts are similar to those in healthy hearts; therefore, healthy hearts were used to as control for cryoinjured hearts. To confirm the downregulation of *col5a1* upon the administration of COL5A1 G2, RNA was extracted from 1–2 pooled hearts, livers, and kidneys of cryoinjured zebrafish.

To detect gene expression, reverse transcription was carried out using a SuperScript® VILO™ cDNA synthesis kit (Invitrogen, Carlsbad, CA, USA). Quantitative PCR was performed with gene specific primers and the LightCycler^®^ 480 SYBR Green I Master (Roche). Glyceraldehyde 3-phosphate dehydrogenase (*gapdh*) and elongation factor 1α 1a (*ef1α1a*) served as reference genes for normalization. RT-qPCR was performed at least three times with three technical replicates for each sample. Primers are listed in Table 1.

**Table 1.** List of primers used in the study

Target	Species	Primer sequences	Reference
*bmpr2a*	Danio rerio	Forward 5´- CCACTGCACAGCCCCTTCAA −3´ Reverse 5´- ACGAGGACAGCAACCATGGAC−3´	This study
*col5a1*	Danio rerio	Forward 5´- GATCCCAACCAGGGCTGCTC−3´ Reverse 5´- GGAGGTGAGTCTGGCCCCTT−3´	This study
*gapdh*	Danio rerio	Forward 5′- CAGGCATAATGGTTAAAGTTGGTA −3′ Reverse 5′- CATGTAATCAAGGTCAATGAATGG −3′	[8]
*ef1α1a*	Danio rerio	Forward 5´- GAGACGCGGCCATTGTGGAA −3´ Reverse 5´- ACCGTCTGACGCATGTCACG −3´	[51]
*ACTA2*	Homo sapiens	Forward 5´- AGCGTGGCTATTCCTTCGTT −3´ Reverse 5´- GCAGTGGCCATCTCATTTTCA −3´	This study
*COL5A1*	Homo sapiens	Forward 5´- AGAAGTCCGAAGGGGCCAGA −3´ Reverse 5´- AGGAGAGCAGTTTCCCACGC −3´	This study
*COL1A1*	Homo sapiens	Forward 5´- TCTGCGACAACGGCAAGGTG −3´ Reverse 5´- GACGCCGGTGGTTTCTTGGT −3´	[22]
*KLF5*	Homo sapiens	Forward 5´- ACGACGCATCCACTACTGCG −3´ Reverse 5´- AGTCGCAGCCTTCCCAGGTA −3´	This study
*AIFM1*	Homo sapiens	Forward 5´- TGGTGCATCAGGGGGCAAAA −3´ Reverse 5´- TGAAGCAGATAACGCGGCCT −3´	This study
*ITGB3*	Homo sapiens	Forward 5´- GCGGCAAGTGTGAATGTGGC −3´ Reverse 5´- TGTAGGGCTCCCCGGTCAAA −3´	This study
*ITGB5*	Homo sapiens	Forward 5´- GGCTGTGGTCGGTAGCATCC −3´ Reverse 5´- AAACTCCCTCCGGTCGTGGA −3´	This study
*ITGAV*	Homo sapiens	Forward 5´- TGTCACCTGGGGCATTCAGC −3´ Reverse 5´- GAGGTGGCCGGACCCGTTTA −3´	This study
*LOX*	Homo sapiens	Forward 5´- CCTGGGAATGGCACAGTTGTCA −3´ Reverse 5´- TGGCCTTCAGCCACTCTCCT −3´	This study
*POSTN*	Homo sapiens	Forward 5´- CGGGACCAAGGCCCAAATGT −3´ Reverse 5´- ATGGGCAAAACTGCTGGGCA −3´	This study
*GAPDH*	Homo sapiens	Forward 5´- TGTTCGACAGTCAGCCGCAT −3´ Reverse 5´- CGCCCAATACGACCAAATCCGT −3´	This study
hsa-miR-145-5p	Homo sapiens	GUCCAGUUUUCCCAGGAAUCCCU	ID YP00204483, Qiagen
hsa-miR-181a-5p	Homo sapiens	AACAUUCAACGCUGUCGGUGAGU	ID YP00206081, Qiagen
hsa-miR-146a-5p	Homo sapiens	UGAGAACUGAAUUCCAUGGGUU	ID YP00204688, Qiagen
hsa-miR-103a-3p	Homo sapiens	AGCAGCAUUGUACAGGGCUAUGA	ID YP00204063, Qiagen

To analyse miRNA expressions in human ventricular samples, RNAs were extracted from snap-frozen LV samples as previously described [51]. Briefly, LV samples were homogenized with OMNI Bead Ruptor for 30 s (6 m/s) in QIAzol reagent (Qiagen), followed by RNA extraction with miRNeasy Mini Kit (Qiagen). Reverse transcription was performed with a miScript II RT kit (Qiagen). Quantitative PCR was performed with a miScript primer assay (Qiagen) and miScript SYBR Green PCR kit (Qiagen). Small Nucleolar RNA, U5G (RNU5G, Catalog #339350, Qiagen) and miR-103a (Qiagen) served as internal controls for normalization. Primers are listed in Table 1.

### Cell culture

Human primary cardiac fibroblasts (HCF, Catalog #6300, ScienCell™ Research Laboratories, Carlsbad, CA, USA) were cultured in poly-L-lysine (Sigma Aldrich, St. Louis, MO, USA) coated flasks. Cells were grown in FGM™^−3^ Cardiac Fibroblast Growth Medium-3 BulletKit™ medium (Lonza, Walkersville, MD, USA) at 37 °C in a 5% CO_2_ atmosphere until 70–90% confluent. For overexpression or silencing of miR-145-5p, HCF cells were transfected with 10 nM hsa-miR-145-5p miRCURY LNA miRNA Mimic (ID YM00470014; 5'GUCCAGUUUUCCCAGGAAUCCCU, Qiagen) or 50 nM hsa-miR-145-5p miRCURY LNA miRNA Inhibitor (ID YI04102423; GGATTCCTGGGAAAACTGGA, Qiagen) using HiPerFect Transfection Reagent (ID 301704, Qiagen) according to the manufacturer’s protocol. Briefly, HCFs were seeded in 6-well plates, followed by addition of mimic/inhibitor–transfection reagent complexes in serum-free medium, and cultured for 48 hours. AllStars Negative Control siRNA (ID 1027280, Qiagen) and miRCURY LNA miRNA Inhibitor Negative Control (ID YI00199006; TAACACGTCTATACGCCCA, Qiagen) served as negative control of mimic and inhibitor, respectively. For knockdown of COL5A1*,* HCF were transfected with 10 nM Antisense LNA™ COL5A1 HS DR1 GapmeR (ID 339511 LG00851813-DDA, A*G*C*T*T*C*A*G*A*G*A*G*T*T*G*A, Qiagen) or Antisense LNA™ COL5A1 HS DR2 GapmeR (ID 339511 LG00851814-DDA, A*G*T*A*T*T*C*A*C*C*A*T*C*T*G*G, Qiagen) using HiPerFect Transfection Reagent for 72 hours. Antisense LNA™ Negative Control A (ID 339515 LG00000002-DDA, A*A*C*A*C*G*T*C*T*A*T*A*C*G*C, Qiagen) and Antisense LNA™ GAPDH (human) Positive Control (ID 339515 LG00000005-DDA, A*G*A*T*T*C*A*G*T*G*T*G*G*T*G*G, Qiagen) were included as negative and positive control, respectively. For co-transfection of miR-145-5p inhibitor and Antisense LNA™ COL5A1 GapmeR, 25 nM miR-145-5p inhibitor together with 5 nM Antisense LNA™ COL5A1 HS DR2 GapmeR were added to the cells. HCFs were exposed to cilengitide at concentrations of 1, 5 or 10 µM for 48 hours to examine the optimal dosage of cilengitide. For co-treatment experiments, cells were incubated with 5 µM cilengitide in combination with 50 nM miR-145-5p inhibitor for 48 hours. All experiments were carried out in triplicates, and the experiments were repeated three times, unless otherwise indicated.

### Luciferase reporter assay

Two potential binding regions for miR-145-5p in the *COL5A1* 3′UTR, CCAAGAACGTGCAATAAATTGGAA (741–764), and ATTTTTTCCTCTCAATATATATAATTGGAC (2264-2293) (miRTarBase-#MIRT053211; https://mirtarbase.cuhk.edu.cn/~miRTarBase/miRTarBase_2019/php/detail.php?mirtid=MIRT053211) were cloned into the dual Luc vector pEZX-MT06 (GeneCopoeia, Rockville, MD). The correct insertion was confirmed by sequencing. The primer sets used for cloning are listed in Table 1. HEK293 cells (a kind gift from Dr. Tiina Rasila, University of Helsinki) were grown in DMEM (Gibco) supplemented with 10% FCS and 100 U/ml penicillin and 100 µg/ml streptomycin (Sigma-Aldrich, St. Louis, MO, USA). The cells were transfected with Luc-*COL5A1*3´UTR constructs together with 20 nM miR-145-5p mimic or mimic control in 96-well plate for 24 hours using HiPerFect Transfection Reagent (Qiagen). Cell lysates were subjected to measurements of firefly and *Renilla* Luc activities using the Dual-luciferase reporter assay kit (Promega, Madison, WI). The firefly signals were normalized using the *Renilla* signals.

### Western blotting

Western blotting was performed as described previously [51]. Briefly, cells were lysed in RIPA buffer (50 mM Tris-HCl, pH 7.4, 150 mM NaCl, 0.25% sodium deoxycholates, 1mM EDTA, 1% NP–40) supplemented with EDTA-free protease inhibitor and phosphatase inhibitor cocktails (Roche, Basel, Switzerland). Protein extracts were resolved on 7.5–10% SDS-PAGE and transferred onto PVDF membranes (Bio-Rad Laboratories, Hercules, CA, USA). Membranes were blocked with EveryBlot Blocking buffer (BioRad) and incubated with the primary antibodies followed by horseradish peroxidase-conjugated or fluorescence-labelled secondary antibodies. The blots were detected using Pierce™ Enhanced Chemiluminescence (ECL) substrate (Pierce Biotechnology, Rockford, IL, USA) of horseradish peroxidase. Quantification was performed using a Gel Doc Image Analyzer (Bio-Rad). Western blotting was performed at least three times. Antibodies are listed in Table 2.

**Table 2:** List of antibodies used in this study

Antibody	Host	Manufacturer	Reference
COL5A1	Mouse	Sigma-Aldrich	WH0001289M1
ɑSMA	Mouse	Dako	M0851
COL1A1	Mouse	DSHB	SP1.D8
vimentin	Rabbit	Cell Signalling	#5741
Integrin β5	Rabbit	Cell Signalling	#4708
Integrin β3	Rabbit	abcam	ab119992
Phospho-Smad2/3	Rabbit	Sigma-Aldrich	SAB4504208
Smad2/3	Rabbit	abcam	ab217553
β-Tubulin	Mouse	Millipore	05–661
HRP-rabbit IgG (H + L)	Goat	Jackson ImmunoResearch	111–035–003
HRP-mouse IgG + IgM (H + L)	Goat	Jackson ImmunoResearch	115–035–044
StarBright Blue 520 –mouse IgG	Goat	BioRad	#12005866
StarBright Blue 700 –rabbit IgG	Goat	BioRad	#12004161
AlexaFluor^®^ 594–mouse IgG	Goat	Invitrogen	# A–11005
Alexa Fluor^®^ 488–rabbit IgG	Goat	Invitrogen	#A–11008

### Immunocytochemistry

HCF cells were cultured on coverslips pre-coated with poly-L-lysine and transfected with miR-145-5p mimic, miR-145-5p inhibitor, or corresponding controls for 48 hours. Cells were fixed in 4% paraformaldehyde in PBS for 10 min at RT and blocked with blocking buffer (10% normal donkey serum, 1% BSA, 0.3% Triton X-100 in PBS), followed by incubation with primary antibodies against αSMA and vimentin overnight at 4°C. The immunoreactivity was detected with AlexaFluor 488 and AlexaFluor 594 labelled secondary antibodies. Nuclei were labelled with DAPI (4`,6-Diamidino-2-Phenylindole) (Molecular Probes, Eugene, OR, USA). Coverslips were mounted in ProLong Diamond antifade mountant (Molecular Probes) and imaged with a Zeiss LSM780 confocal microscope (Carl Zeiss Microscopy GmbH, Jena, Germany). ImageJ 1.53f51 software was used to process images and to quantify the αSMA-positive cells.

### Wound healing assay

HCF cells were seeded in a 48-well plate and transfected for 48 hours. A vertical scratch was made with a 200 μL pipette tip followed by extensive washes with PBS to remove the detached cells. Subsequently, a complete growth medium was added. At 0, 4, and 24 hours post-treatment, images were captured using an Invitrogen EVOS™ microscope (Thermo Scientific) and analysed with Image J. The wound closure was calculated at 4 hours post-treatment.

### Real‑time cell proliferation and migration assay

Proliferation of HCF cells transfected with miR-145-5p mimic, miR-145-5p inhibitor, or corresponding controls was measured using an xCELLigence Real-Time Cell Analysis (RTCA) instrument with E-16 plates (Agilent Technologies, San Diego, CA, USA). Transfected cells for 48 hours were seeded at a density of 1 × 10^4^ cells/well on poly-L-lysine coated microtiter plates with gold microelectrode biosensors (E-plate 16, Agilent Technologies). Cell density was detected by monitoring changes in electrical impedance at 15-min intervals for 12 hours followed by 30-min intervals for 12 hours with RTCA Software 1.2 (Agilent Technologies). Cell Index (CI) that is related to cellular density was normalized to that at 2 hours. Calculated CI values were plotted on a line graph. Each experimental condition was tested in triplicate. The effect of COL5A1 downregulation on HCF proliferation was performed as described above except that HCF cells were transfected with Antisense LNA™ COL5A1 HS DR2 GapmeR or Negative Control for 48 hours prior to the xCELLigence assays. To assess the effect of cilengitide alone or in combination with the miR-145-5p inhibitor on HCF proliferation, HCFs were treated with 5 µM cilengitide alone or together with 50 nM miR-145-5p inhibitor for 48 hours prior to seeding onto fibronectin-coated (Sigma-Aldrich) E-16 plates. The cell density was monitored for 24 hours.

Migration of HCF cells transfected with Antisense LNA™ COL5A1 HS DR2 GapmeR or Negative Control was measured using an xCELLigence RTCA DP instrument with CIM-plate 16 according to instruction (Agilent Technologies). Transfected cells for 48 hours were suspended and seeded at a density of 2 × 10^4^ cells/well into upper chamber of CIM-plate 16 containing low FBS (1%) FGM™^−3^ media. The wells of the lower chamber were loaded with FGM™^−3^ complete media. As the cells migrated towards the chemoattractant across microelectronics sensors, the impedance was measured for 24 hours at 15-min intervals. All experiments were performed in triplicate and repeated three times. However, the proliferation assays of HCF treated with cilengitide alone or in combination with miR-145-4p inhibitor were repeated twice.

### Histology

Acid fuchsin orange G (AFOG, Sigma-Aldrich, Saint Louis, MO, USA) staining was performed to detect fibrotic tissue, as previously described [52]. Briefly, paraffin sections were dewaxed and post-fixed in Bouin´s solution for 60 minutes at 60 °C in a water bath, then stained with Weigert´s iron haematoxylin and AFOG solution. The infarct region and ventricle size were measured using ImageJ. Fibrin and collagen stains in the infarct region were analysed using QuPath 0.6.0 software.

### Statistical analysis

The data were analysed using Prism software (version 10.3.0; GraphPad, San Diego, CA, USA) and are presented as mean ± SD. One-way ANOVA with Tukey's adjustment or two-way ANOVA with Dunnett´s adjustment was used to calculate differences between more than two groups with normally distributed data. A two-sample, unpaired, two-tailed test was performed to compare two groups with normally distributed data. Data that did not pass the normality and lognormality tests were analysed with the Mann–Whitney test to compare the difference between two groups, or the Kruskal–Wallis followed by Dunn's test for three groups. Statistical significance was set at *p*<0.05.

## Results

### Cardiac recovery and transcriptomic alterations following cryoinjury in adult zebrafish

**Fig. 1:**
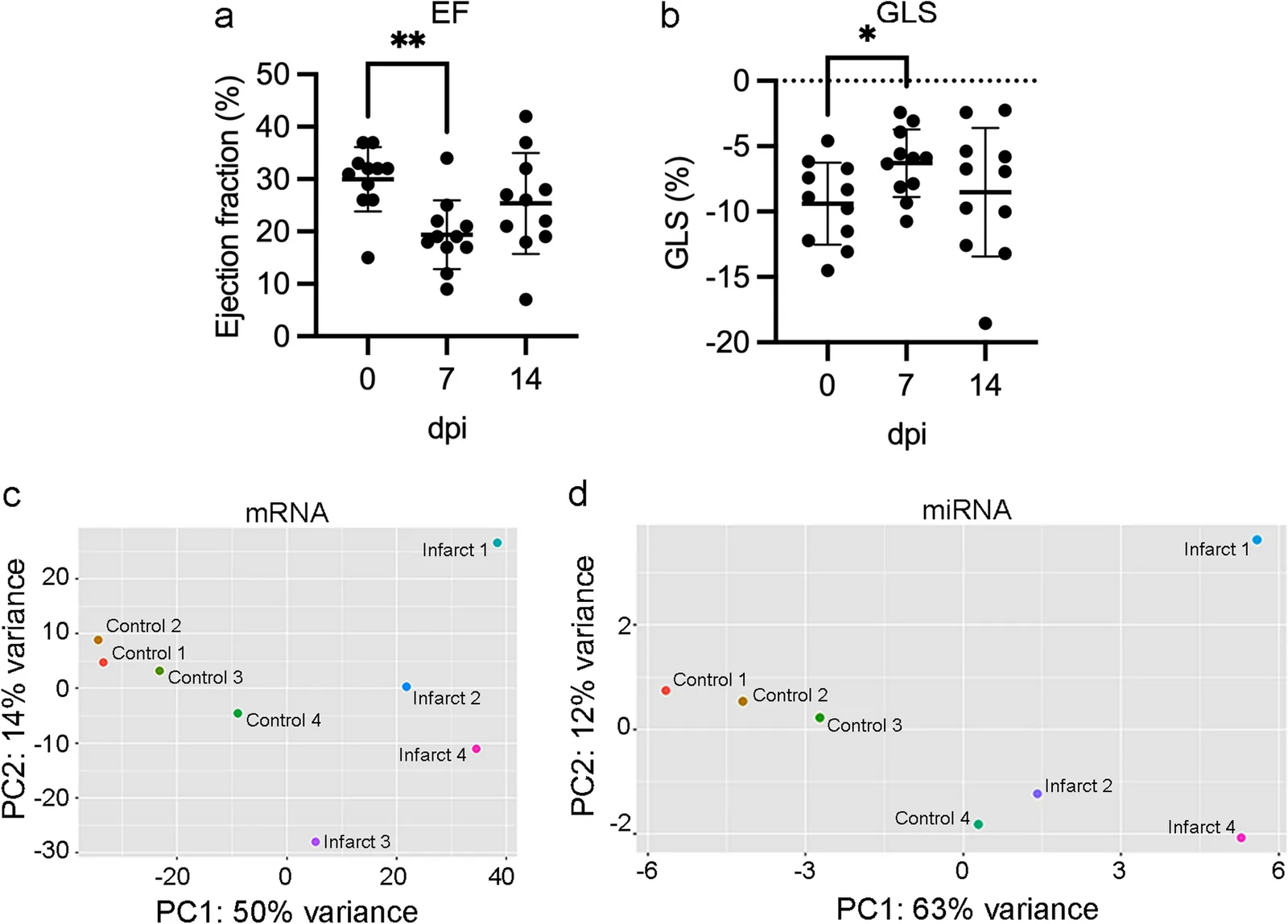
Cryoinjury impairs cardiac function and alters transcriptome profiles in zebrafish hearts. **a-b** Cryoinjury impairs cardiac contractility at 7 dpi as manifested by the reduction in EF (**a**) and GLS (**b**) compared to baseline. The defect appears alleviated at 14 dpi. *n* = 11. **c-d** Principal component analyses of the mRNAome (**c**) and the miRNome (**d**). Data are presented as mean ± SD. One-way ANOVA with Tukey adjustment for multiple comparisons. * *p* < 0.05, ** *p* < 0.01

Cryoinjury in adult zebrafish led to a significant reduction in EF (*p*=0.003) (Fig. 1a) and a slight decrease in GLS (*p*=0.05) (Fig. 1b) at 7 dpi compared with baseline. By 14 dpi, neither EF nor GLS differed significantly from baseline (Fig. 1a, b), indicating partial recovery of cardiac contractility.

A prior study showed that early post-cryoinjury responses (1–7 dpi) were characterized mainly by cell proliferation and immune activation, whereas intermediate phases (14–45 dpi) were marked by ECM remodelling and recovery of cardiac function [27]. To identify molecular pathways associated with cardiac fibrotic scar remodelling, mRNA and miRNA were isolated from healthy or cryoinjured hearts at 14 dpi and subjected to high-throughput transcriptome analysis. Principal component analyses (PCA) of the mRNAome showed clear separation between healthy and injured hearts along PC1, which accounted for 50% of the variance (Fig. 1c). Similarly, PCA of the miRNome revealed distinct clustering corresponding to healthy and cryoinjured hearts, with PC1 explaining 63% of the variance (Fig. 1d). These data indicate that both mRNA and miRNA expression profiles are substantially altered during the regenerative phase following cryoinjury.

### Transcriptomic analysis reveals extracellular matrix remodelling in regenerating zebrafish hearts

**Fig. 2:**
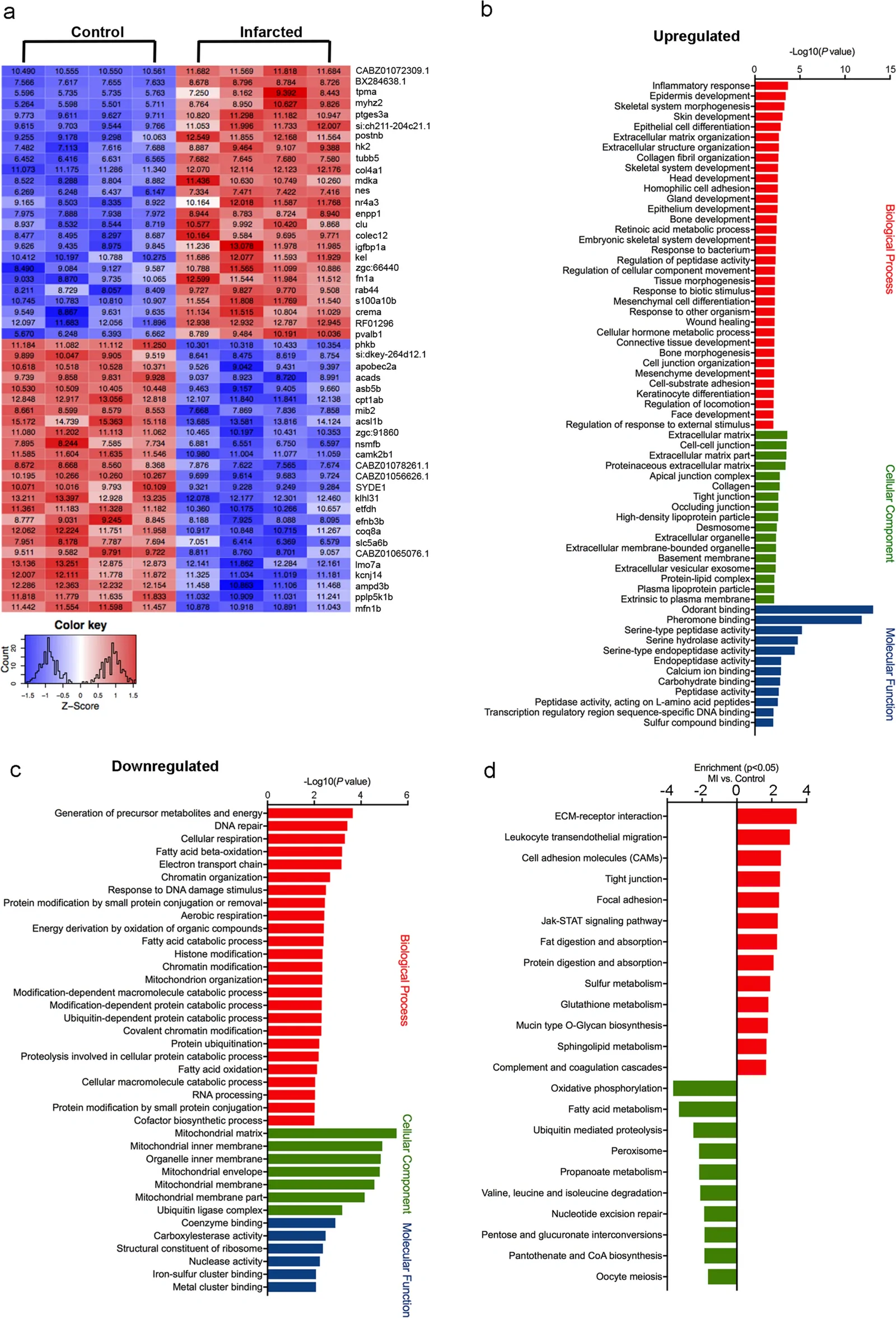
Differentially expressed genes and significantly modulated molecular pathways in cryoinjured hearts at 14 dpi. **a** Heatmap shows the top 25 upregulated and downregulated genes ordered by adj. *p*-value in cryoinjured hearts at 14 dpi. Red colour represents upregulated genes while blue indicates downregulated genes. **b-c **Gene set enrichment analysis using GO categories indicates positive enrichment of GO-categories (**b**) and negative enrichment of GO-categories (**c**) in cryoinjured hearts. **d** Gene set enrichment analysis using the KEGG pathway browser. Red indicates positive enrichment; green indicates negative enrichment

We identified 4379 differentially expressed genes (DEGs), of which 896 genes were significantly upregulated, and 154 genes downregulated with nominal *p*-value < 0.05 in regenerating zebrafish hearts (Table S1). Scaled heatmap demonstrates the top 25 upregulated and downregulated genes ordered by Benjamini-Hochberg adjusted *p*-values (adj. *p*-value) in the regenerating hearts (Fig. 2a). The most strongly upregulated genes included two unknown genes (CABz01072309.1 and BX284638.1), followed by *tropomyosin alpha (tpma)* and *myosin heavy polypeptide 2* (*myhz2*), both involved in muscle contraction. On the other hand, the most downregulated gene was *phosphorylase kinase beta* (*phkb*), associated with cellular energy metabolism (Fig. 2a).

To gain insights into significantly modulated biological pathways, we performed GSEA. GO categories illustrated enrichment for genes related to inflammatory response and extracellular matrix (ECM) organization in cryoinjured hearts (Fig. 2b). On the other hand, genes associated with generation of precursor metabolites and mitochondrial matrix organization were downregulated in cryoinjured hearts (Fig. 2c). KEGG pathway analyses highlighted significant enrichment in ECM-receptors interaction, ECM organization, leukocyte trans-endothelial migration, and cell-cell adhesion (Fig. 2d; Fig. S1). Pathways involved in oxidative phosphorylation and fatty acid metabolism were notably downregulated (Fig. 2d; Fig. S2). Our data suggest the activation of molecular pathways that promote inflammation and ECM remodelling essential for tissue repair and the suppression of metabolic and mitochondrial functions related to energy production in cryoinjured hearts at 14 dpi.

### Alterations in miRNA expression profiles during zebrafish cardiac regeneration

**Fig. 3:**
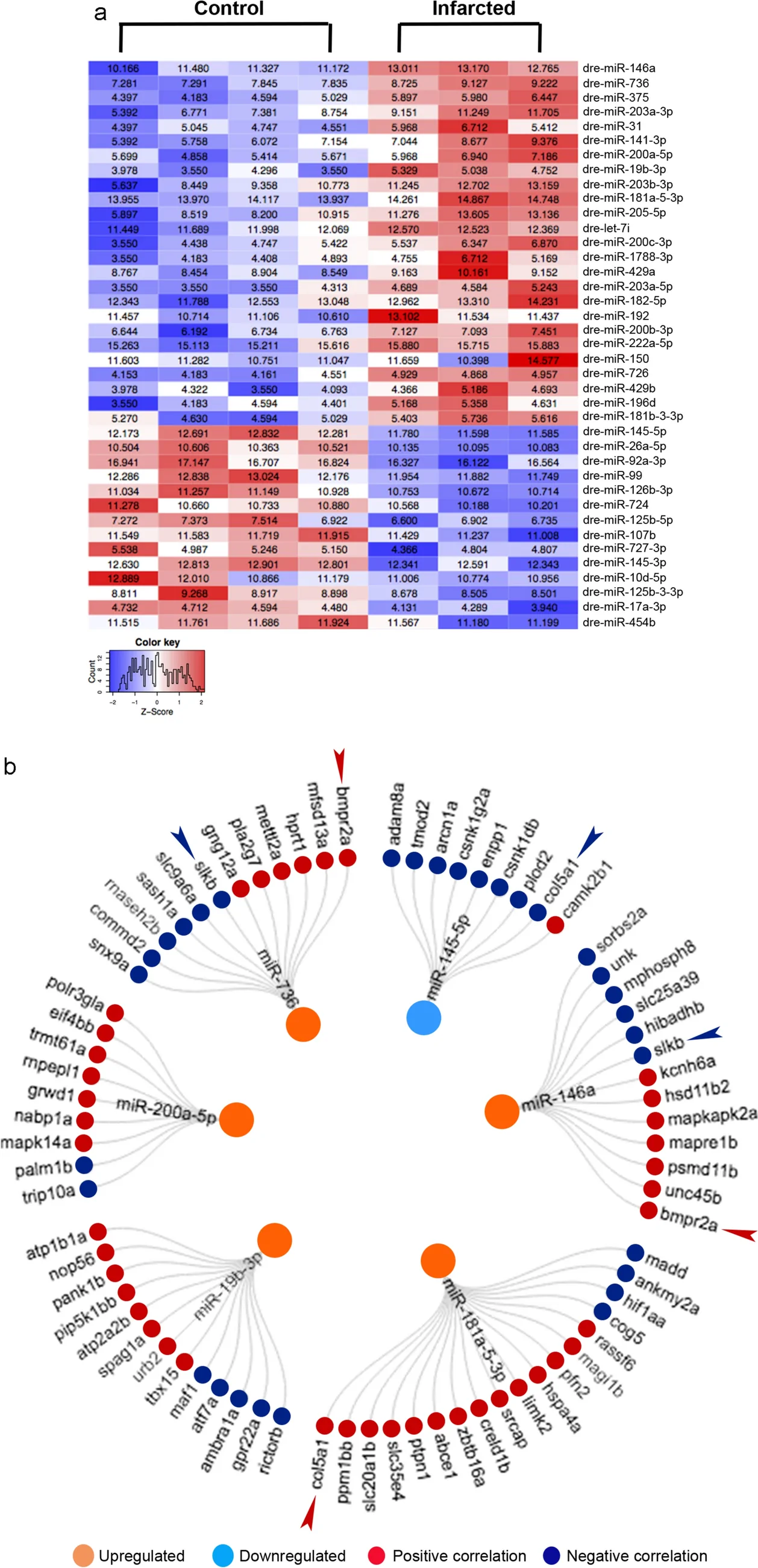
Differentially expressed miRNAs in cryoinjured hearts at 14 dpi and their predicted targets. **a **Heatmap shows the top 25 upregulated and 14 downregulated miRNAs ordered by adj. *p*-value (adj. *p*-value < 0.05) in cryoinjured hearts at 14 dpi. Red colour represents upregulated miRNAs while blue indicates downregulated miRNAs. **b** Predicted targets for the differentially expressed miRNAs (adj. *p*-value < 0.05) in cryoinjured hearts. The predicated targets are also differentially expressed in cryoinjured hearts (adj. *p*-value < 0.05). Orange colour represents the upregulated while light blue indicates downregulated miRNAs. Red colour shows positive correlation between miRNA and their target genes; dark blue indicates negative correlation. Arrowhead points the target genes of multiple miRNAs

We detected 290 mature miRNAs, of which 34 were upregulated and 14 downregulated in cryoinjured hearts at 14 dpi with *p*-value < 0.05 as compared to control hearts (Table S2). The top 25 upregulated and 14 downregulated miRNAs in the regenerating hearts are shown, ordered by *p-*values, as a heat map (Fig. 3a). Among these miRNAs, ten were found to be significantly upregulated and one downregulated with adj. *p*-value <0.05 (Table S2). miR-146a was the most upregulated and miR-145-5p the most downregulated miRNA in cryoinjured hearts (Fig. 3a). Our data indicate that cardiac cryoinjury induces alteration of miRNome, supporting a role for miRNAs in regulation of gene expression during cardiac regeneration.

### Differential expression of miRNAs and predicted targets in regenerating zebrafish heart and failing human heart

Given that miRNAs negatively regulate mRNA expression and appear to be essential regulators during cardiac generation, we hypothesized that differentially expressed miRNAs would modulate pathways associated with cardiac regeneration. We, therefore, performed a theoretical prediction of miRNA–mRNA interactions for eleven miRNAs with an adj. *p*-value <0.05 using TargetScanFish 6.2 (Table S2). The target genes with a total context plus score in the top 25 percentile according to Target Scan predictions were selected for calculating the Pearson correlation with the corresponding miRNAs. We selected the DEGs (adj. *p*-value < 0.05) with a significant correlation (Pearson´s r *p*<0.05) to miRNAs and excluded the miRNAs with fewer than five DEGs in cryoinjured hearts. miR-181a-5-3p appeared to target the largest number of DEGs in the heart (Fig. 3b). Interestingly, *procollagen Vα1* (*col5a1*) displays a positive correlation with the upregulated miR-181a-5-3p and a negative correlation with the downregulated miR-145-5p (Fig. 3b). Ultimately, this resulted in an upregulation of *col5a1.* In addition, *bone morphogenetic protein receptor II a* (*bmpr2a*) is positively correlated with miR-146a and miR-736 that are markedly upregulated in cryoinjured hearts (Fig. 3b). Conversely, *STE20-like kinase b* (*slkb)* exhibits a negative correlation with these two miRNAs (Fig. 3b). The data suggest that cryoinjury stimulates the expression of miR-146a and miR-736, which consequently upregulate *bmpr2a* and downregulate *slkb.* Our data indicate multiple differentially expressed miRNAs can coordinate the regulation of common target genes and thereby influence key molecular pathways during cardiac regeneration.

**Fig. 4:**
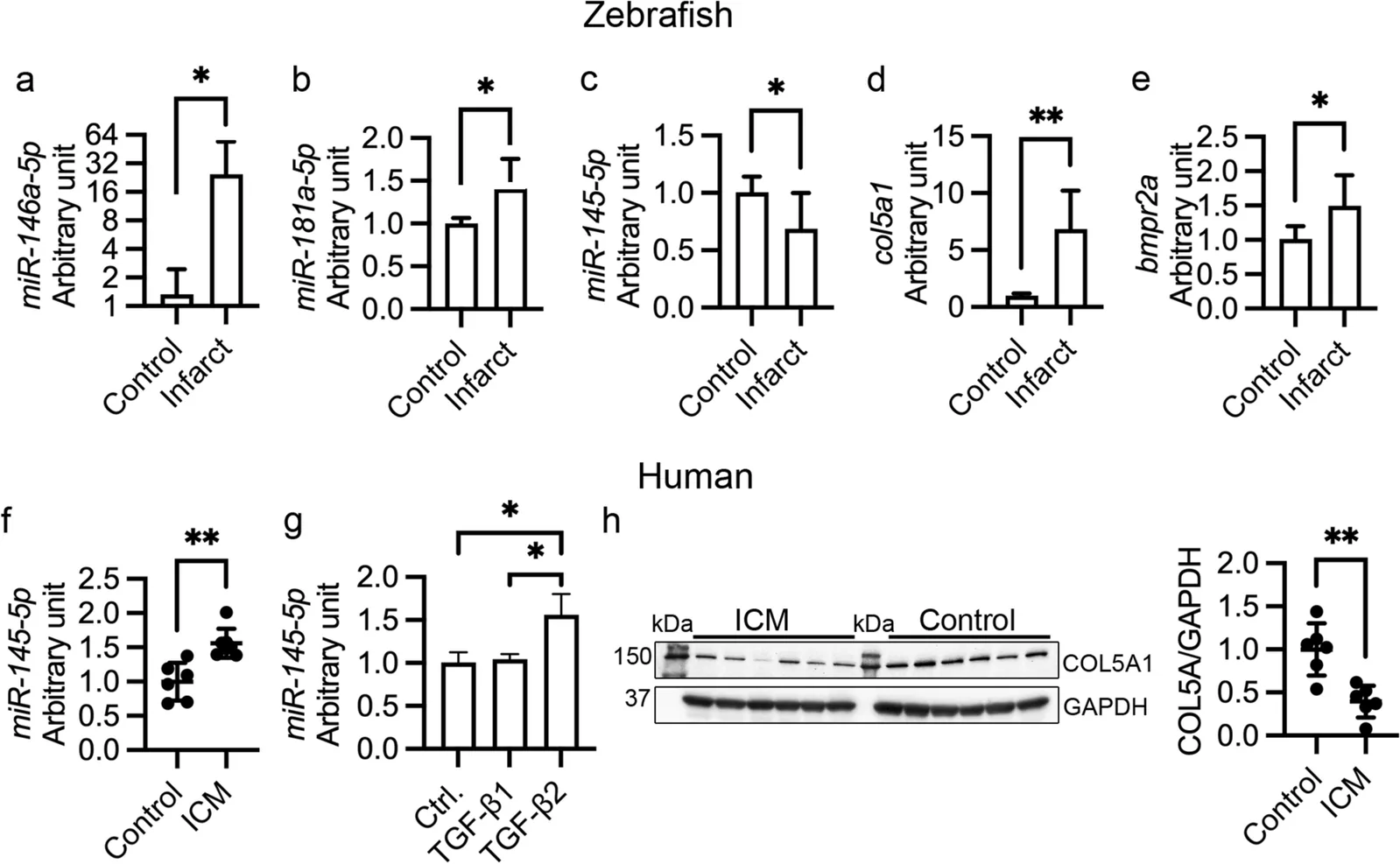
Validation of the most differentially expressed miRNAs and genes in cryoinjured hearts. **a-c **RT-qPCR analyses of the expression of miR-146a-5p (**a**), miR-181a-5p (**b**), and miR-145-5p (**c**) in zebrafish cryoinjured hearts compared to healthy controls. The results are presented as fold change. **d-e** RT-qPCR analyses of the expression of *bmpr2a* (**d**) and *col5a1* (**e**) in zebrafish cryoinjured hearts compared to healthy controls. The results are presented as fold change. *bmpr2a, bone morphogenetic protein receptor II a; col5a1, procollagen Vα1. *RNA was extracted from 3 to 4 pooled hearts. The number of RNA samples used for RT-qPCR analyses: control* n* = 3; infarct *n* = 3. **f-g **RT-qPCR analysis of miRNA-145-5p expression in the LV of ICM patients (**f**) and human primary cardiac fibroblast (HCF) treated with TGF-β1 or TGF-β2 for 24 hours (**g**) compared to respective controls. Controls *n* = 6; ICM *n* = 7. **h **Representative immunoblot and quantification of COL5A1 in the LV of ICM patients compared to controls. Controls *n* = 6; ICM *n* = 6. Data are presented as mean ± SD. Two-sample *t*-test. One-way ANOVA with Tukey adjustment for multiple comparisons. * *p* < 0.05, ** *p* < 0.01

To validate the results, we performed RT-qPCR to determine the most differentially expressed miRNAs in cryoinjured hearts. Consistent with the RNA-seq data, miR-146a-5p (*p*=0.03) and miR-181a-5p (*p*=0.02) were markedly upregulated, while miR-145-5p (*p*=0.04) was substantially downregulated in cryoinjured hearts (Fig. 4a-c). We quantitively analysed the expression level of the target genes, *bmpr2a and col5a1.* As expected*, col5a1* (*p*=0.002) and *bmpr2a* (*p*=0.04) were significantly upregulated in cryoinjured hearts (Fig. 4d, e).

As miR-145-5p is the most downregulated microRNA and is inversely correlated with *col5a1* in zebrafish regenerating hearts, we investigated the expression of miR-145-5p and COL5A1 in LV of ICM patients undergoing heart transplantation compared with healthy controls to explore the potential involvement of miR-145-5p in adverse cardiac remodelling in humans. In contrast to zebrafish, miR-145-5p (*p*=0.002) was significantly upregulated in the LV of ICM patients (Fig. 4f). We also observed a significant increase in the expression level of miR-145-5p (*p*=0.01) in HCF following treatment with transforming growth factor-beta 2 (TGF-β2) for 24 hours (Fig. 4g), consistent with the finding that TGF-β induces miR-145-5p expression in various cell types [33, 48]. Furthermore, we detected a reduced protein level of COL5A1 (*p*=0.004) in the LV of ICM patients (Fig. 4h). Thus, miR-145-5p and COL5A1 displayed opposite expression patterns in the regenerating zebrafish heart versus the decompensated human heart.

### Regulatory role of miR-145-5p in cardiac fibroblast dynamics

**Fig. 5:**
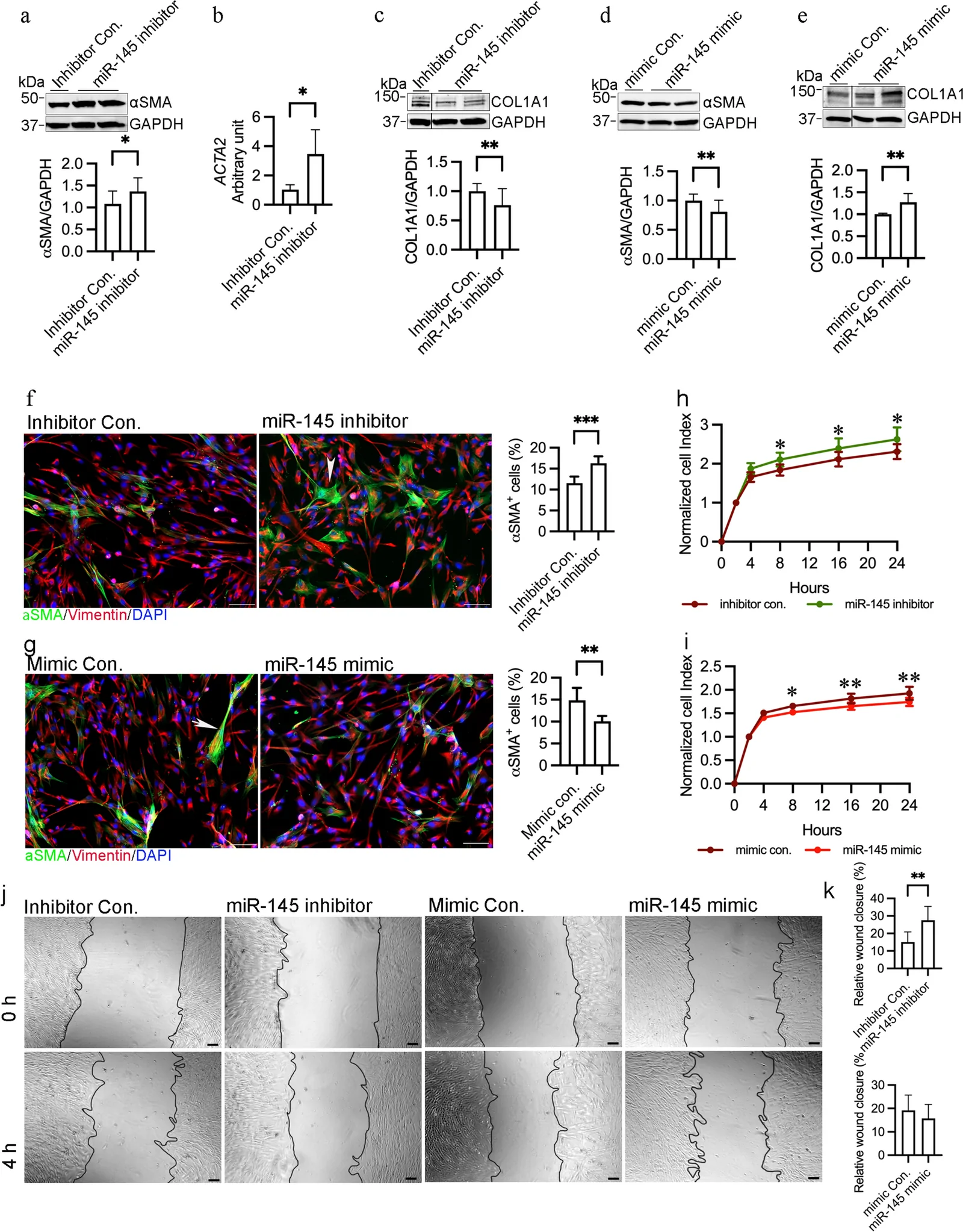
miR-145-5p regulates collagen expression and cardiac fibroblasts differentiation, proliferation, and migration. **a** Representative immunoblot and quantification of αSMA in HCF either treated with miR-145-5p inhibitor or inhibitor control. **b **RT-qPCR analysis of *ACTA2* transcript in HCF either treated with miR-145-5p inhibitor or inhibitor control. **c **Representative immunoblot and quantification of COL1A1 in HCF either treated with miR-145-5p inhibitor or inhibitor control. **d **Representative immunoblot and quantification of αSMA in HCF either treated with miR-145-5p mimic or mimic control. **e **Representative immunoblot and quantification of COL1A1 in HCF either treated with miR-145-5p mimic or mimic control. **f-g **Representative immunocytochemical staining of αSMA (in green) and vimentin (in red) in HCF treated with miR-145-5p inhibitor (**f**) or miR-145-5p mimic (**g**) and their respective controls. Graph indicates the quantification of αSMA^+^ cells relative to DAPI counts. Arrow indicates αSMA^+^ fibroblast. Scale bar: 50 µm. **h-i **Real-time proliferation profiles of HCF transfected with miR-145-5p inhibitor (**h**), miR-145-5p mimic (**i**), or corresponding controls. Prior to the xCELLigence assays, cells were transfected for 48 hours, and cell density was monitored for 24 hours. Cell Index was normalized to that at 2 hours. **j **Representative images of wounds immediately after wounding (0 hour) and at 4 hours. Prior to wounding, transfected HCF were grown for 48 hours to confluence. Scale bar: 200 µm. **k **Graph bar represents the relative wound closure area at 4 hours to 0 hour. Data are presented as mean ± SD. Two-sample *t*-test. * *p* < 0.05, ** *p* < 0.01, *** *p* < 0.001

During zebrafish cardiac regeneration, fibrotic scar resolution is an essential process that is largely absent in the context of human myocardial infarction [43]. By around 14 dpi, the fibrotic scar begins to decrease, a process that coincides with extensive remodelling of ECM. Accordingly, we explored whether a low level of miR-145-5p is associated with cardiac ECM remodelling. To achieve this, we inhibited or overexpressed miR-145-5p in HCF. Inhibition of miR-145-5p resulted in increased expression of αSMA at both the protein (Fig. 5a) and mRNA levels (Fig. 5b), while it led to a decrease of COL1A protein level (Fig. 5c). In contrast, overexpression of miR-145-5p reduced the protein level of αSMA (Fig. 5d) and increased that of COL1A (Fig. 5e). By immunocytochemistry using αSMA and vimentin antibodies, we observed more αSMA-positive cells when inhibiting miR-145-5p (Fig. 5f) and fewer αSMA-positive cells when overexpressing miR-145-5p (Fig. 5g), suggesting that miR-145-5p drives fibroblasts towards a collagen-producing phenotype while suppressing myofibroblast differentiation.

The observation of miR-145-5p negatively regulating myofibroblast differentiation prompted us to investigate the proliferation and migration of HCF in response to treatment with miR-145-5p mimic or inhibitor. Consistent with the activation of myofibroblasts, Real-Time Cell Analysis indicated that inhibition of miR-145-5p stimulated cell proliferation (Fig. 5h), while overexpression of miR-145-5p retarded it (Fig. 5i). We further performed wound healing experiments. The wound closure area relative to initial wound area after 4 hours was larger in the case of miR-145-5p inhibition (Fig. 5j and k), while it tended to be smaller in the case of miR-145-5p overexpression (Fig. 5j and k), implying that inhibition of miR-145-5p accelerated cell migration. These results suggest a role of miR-145-5p in regulation of fibroblast proliferation and migration.

### Identification of COL5A1 as a direct target gene of miR-145-5p

**Fig. 6:**
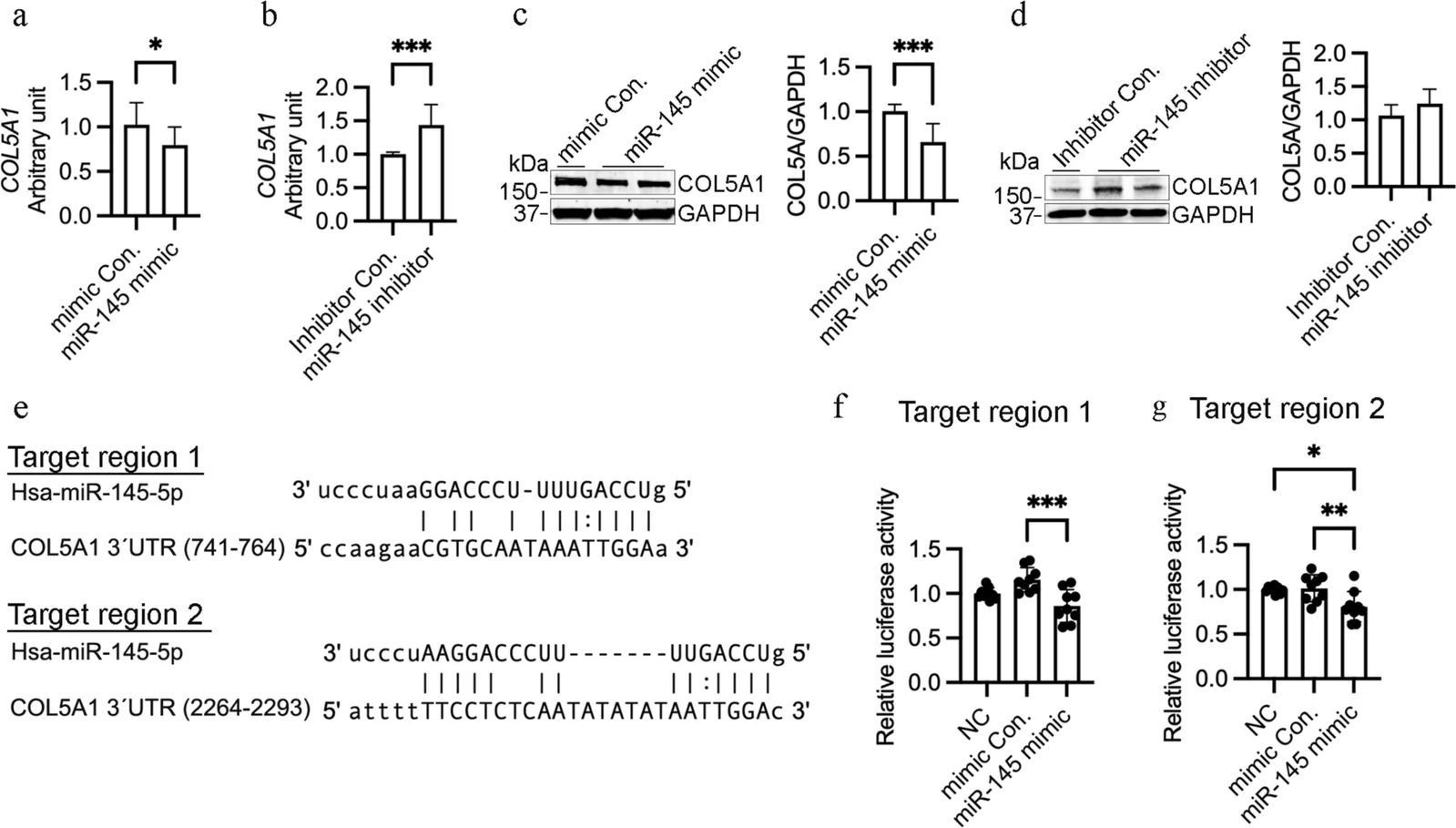
Identification of COL5A1 as a target gene of miR-145-5p. **a-b **RT-qPCR analyses of *COL5A1* expression in HCF either treated with miR-145-5p mimic (**a**) or inhibitor (**b**) compared with relevant controls. **c-d **Representative immunoblot and quantification of COL5A1 in HCF either treated with miR-145-5p mimic (**c**) or inhibitor (**d**) compared with relevant controls. **e, **Schematic presentations of the *COL5A1* 3′UTR with predicted miR-145-5p binding sites. **f-g **The relative firefly luciferase activity was normalized to the Renilla luciferase signal for each sample. HEK cells were transfected with pEZX-MT06-COL5A1-3UTR carrying targeting region 1 (**f**) or region 2 (**g**) either alone or with mimic control, or miR-145-5p mimic, for 24 hours. Data are presented as mean ± SD. Two-sample *t*-test. One-way ANOVA with Tukey adjustment for multiple comparisons. * *p* < 0.05, ** *p* < 0.01, *** *p* < 0.001

The negative correlation between miR-145-5p and COL5A1 observed in cryoinjured zebrafish and ICM patient hearts prompted us to investigate a potential regulatory relationship between miR-145-5p and COL5A1. Overexpression of miR-145-5p downregulated *COL5A1* (Fig. 6a). In addition, Krueppel-like factor 5 (*KLF5*), a transcription factor identified as a miR-145-5p target [54], was also downregulated (Fig. S3a). Another target, *apoptosis-inducing factor mitochondria associated 1* (*AIFM1*) [59], showed a tendency toward decrease upon overexpression of miR-145-5p (Fig. S3b). In contrast, inhibition of miR-145-5p lead to upregulation of *COL5A1* (Fig. 6b) as well as *KLF5* and *AIFM1* (Fig. S3c and d). In line with the transcript level, the protein level of COL5A1 was also decreased when overexpressing miR-145-5p (Fig. 6c), while it showed a tendency toward increase when inhibiting miR-145-5p (Fig. 6d). To validate that *COL5A1* mRNA is a direct target of miR-145-5p, we performed a dual luciferase assay using pEZX-MT06 constructs containing either *COL5A1* 3´UTR (741–764) or (2264–2293) binding sites for miR-145-5p (Fig. 6e). Luciferase activity was significantly reduced in HEK293T cells co-transfected with either construct and miR-145-5p mimic (Fig. 6f, g). No effect on luciferase activity was detected after co-transfection with mimic control (Fig. 6f, g). These results indicate direct binding of miR-145-5p to the *COL5A1* 3ÚTR.

### miR-145-5p regulates human cardiac fibroblasts partially via targeting COL5A1

**Fig. 7:**
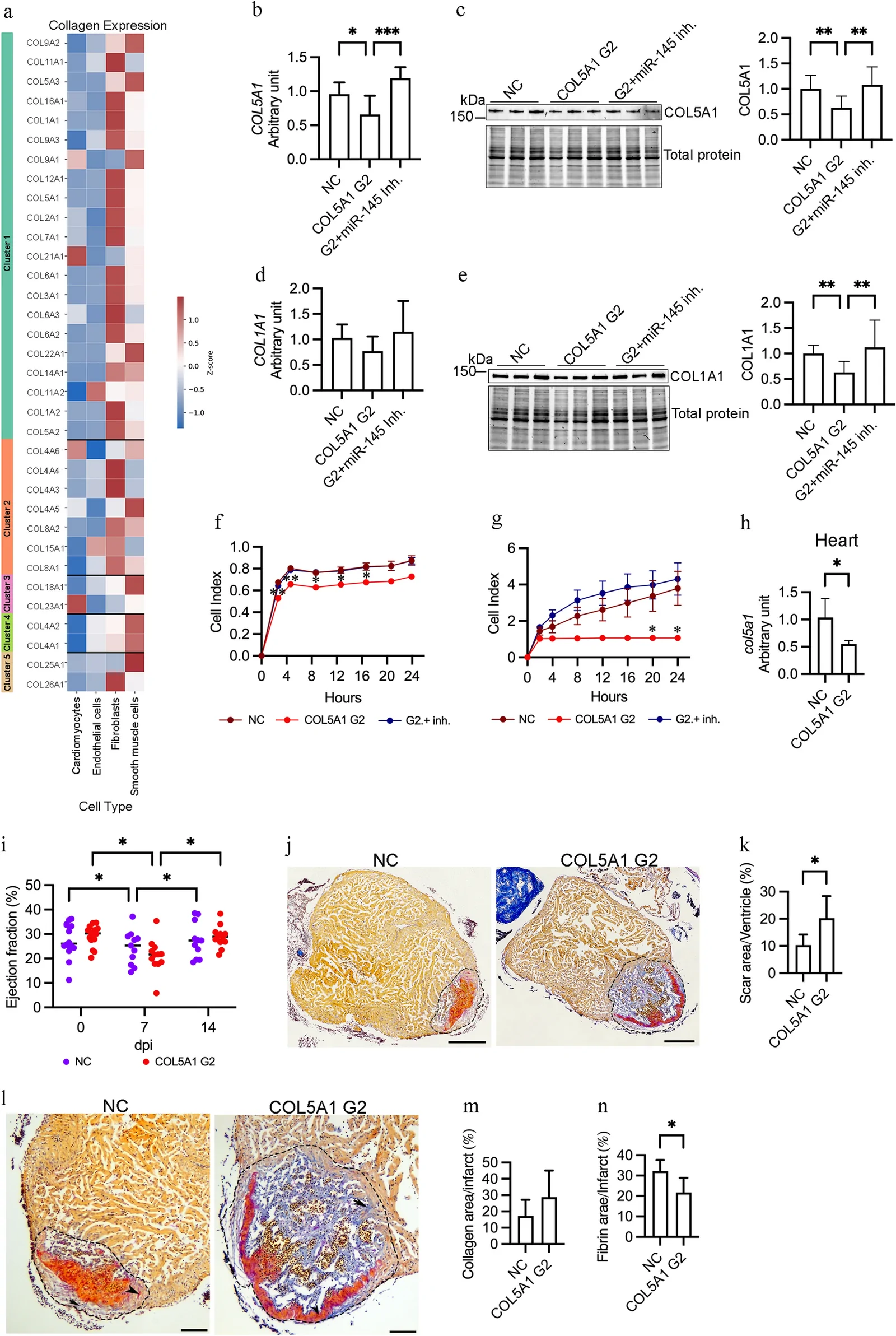
COL5A1 regulates collagen composition, fibroblast dynamics, and cardiac fibrotic scar resolution. **a **Cytoscape analysis of collagen network and their distribution in cardiac cells. Red colour shows higher relative expression level; blue indicates lower level; grey indicates undetected gene in the source data. **b **RT-qPCR analysis of *COL5A1* expression in HCF either treated with COL5A1 GapmeR DR2 (COL5A1 G2) alone or together with miR-145-5p inhibitor (miR-145 inh.), compared to negative control (NC). **c **Representative immunoblot and quantification of COL5A1 in HCF either treated with COL5A1 G2 alone or together with miR-145-5p inhibitor, compared to negative control. **d **RT-qPCR analysis of *COL1A1* expression in HCF either treated with COL5A1 G2 alone or together with miR-145-5p inhibitor, compared to negative control. **e **Representative immunoblot and quantification of COL1A1 in HCF either treated with COL5A1 G2 alone or together with miR-145-5p inhibitor, compared to negative control. **f **Real-time proliferation profiles of HCF transfected with COL5A1 G2 alone or together with miR-145-5p inhibitor, compared to negative control. **g **Real-Time migration profiles of HCF transfected with COL5A1 G2 alone or together with miR-145-5p inhibitor, compared to negative control. **h **RT-qPCR analyses of the expression of *col5a1* in cryoinjured hearts of zebrafish injected with COL5A1 G2 compared with those injected with NC. RNA was extracted from 1 to 2 pooled hearts. The number of RNA samples used for RT-qPCR analyses: NC* n* = 3; COL5A1 G2 *n* = 3. **i **Cryoinjury leads to a significant decline in EF in both COL5A1 G2- and NC-injected zebrafish at 7dpi, but not at 14 dpi compared with baseline. NC, baseline *n* = 16, 7 and 14 dpi *n* = 11; COL5A1 G2, baseline *n* = 16, 7 and 14 dpi *n* = 12. **j **Representative images of acid fuchsin orange G (AFOG) staining of ventricular sections showing fibrotic scars at 14 dpi. Outline indicates scar region. Scale bar: 200 µm. **k **Quantification of scar areas relative to ventricles at 14 dpi. NC, *n* = 6; COL5A1 G2, *n* = 5. Two sections from each heart were stained and analysed. l Representative images of AFOG staining showing accumulation of collagen (blue) and fibrin (red) in the scar regions (outline) at 14 dpi. Arrow indicates collagen. Arrowhead represents fibrin. Scale bar: 75 µm. **m-n **Quantification of accumulated collagen (**m**) and fibrin (**n**) in scar region at 14 dpi. NC, *n* = 6; COL5A1 G2, *n* = 5. Two sections from each heart were analysed. Data are presented as mean ± SD. Two-sample *t*-test. One-way ANOVA with Tukey adjustment or two-way ANOVA with Dunnett adjustment for multiple comparisons. * *p* < 0.05, ** *p* < 0.01, *** *p* < 0.001

To investigate the interaction of COL5 with the collagen network and its expression pattern in cardiac cells, we performed a protein-protein interaction analysis of all detected COL genes in RNA-seq dataset derived from hearts of ICM patients. COL5A1-3 interact closely with COL1A1/2 and COL3A1, and form a core cluster with COL1A1/2, COL2A1, COL3A1 and COL11A1/2 (Fig. S3e and Fig. 7a). The expression patterns of collagen genes in cardiac cells show that *COL5A1-2*, *COL1A1-2*, and *COL3A1* are strongly expressed in fibroblasts (Fig. 7a), while *COL5A3* is more prominently distributed in smooth muscle cells than in fibroblasts (Fig. 7a).

We next investigated whether miR-145-5p regulates cardiac fibroblasts via targeting *COL5A1.* We transfected HCF with either *COL5A1* GapmeR DR1 or DR2 (COL5A1 G1 or G2) and observed significant downregulation of COL5A1 with DR2 at both transcript and protein levels after 72 hours (Fig. S4a, b), but not with DR1. Moreover, the downregulation of COL5A1 at both transcript and protein levels by COL5A1 G2 could be rescued by inhibiting miR-145-5p (Fig. 7b, c), further confirming that miR-145-5p targets COL5A1, or modulates its regulatory network. Interestingly, we observed a trend toward decreased transcript abundance and a significantly decreased protein level of COL1A1 upon downregulation of COL5A1, which could be rescued by inhibition of miR-145-5p (Fig. 7d, e). However, COL5A1 G2 had no significant effect on αSMA (Fig. S4c, d). The results suggest that COL5A1 can influence collagen composition and potentially modify fibrotic repair.

We further investigated the proliferation and migration of cardiac fibroblasts in response to treatment with COL5A1 G2. Real-Time Cell Analysis showed that downregulation of COL5A1 retarded cell proliferation, which could be rescued by inhibition of miR-145-5p (Fig. 7f). Furthermore, downregulation of COL5A1 alone suppressed cell migration, whereas simultaneous inhibition of COL5A1 and miR-145-5p abolished this effect (Fig. 7g). Our data indicate that miR-145-5p regulates collagen composition and fibroblast dynamics partially via targeting *COL5A1*.

### Downregulation of COL5A1 attenuates cardiac scar resolution in adult zebrafish

We next explored whether the downregulation of *col5a1* with the COL5A1 GapmeR G2 affects cardiac fibrotic scar resolution in zebrafish. To verify the efficacy of COL5A1 G2 in zebrafish, we quantitatively analysed the expression level of *col5a1* in hearts, livers, and kidneys at 14 dpi by RT-qPCR. *col5a1* was markedly downregulated in cryoinjured hearts of zebrafish injected with COL5A1 G2 compared with those injected with NC (Fig. 7h), but showed no significant alteration in livers and kidneys (Fig. S4e, f), indicating that COL5A1 G2 is more stable in hearts than in the metabolic clearance organs, liver and kidney. As expected, cryoinjury led to a significant decline in EF in both COL5A1 G2- and NC-injected zebrafish at 7dpi (Fig. 7i). Moreover, zebrafish injected with COL5A1 G2 exhibited a more pronounced decline in EF with a 27.6% decrease from baseline, compared with an 11.5% decrease in NC-injected zebrafish at 7 dpi, but this difference did not reach statistical significance. At 14 dpi, neither group displayed a significant change in EF from baseline (Fig. 7i). However, histological staining revealed that the area of fibrotic scar tissue was significantly larger in hearts with *col5a1* downregulation than in the control group (Fig. 7j, k). In addition, an 11.58% increase in collagen accumulation was observed in the scar area of *col5a1-*knockdown hearts compared with controls (Fig. 7l, m), although the change did not reach statistical significance. Notably, *col5a1-*knckdown hearts displayed a profound decrease in fibrin in the scar area in comparison with controls. Taken together, downregulation of *col5a1* attenuated cardiac fibrotic scar resolution in zebrafish.

### miR-145-5p potentially regulates integrin-mediated pathways in human cardiac fibroblast

**Fig. 8:**
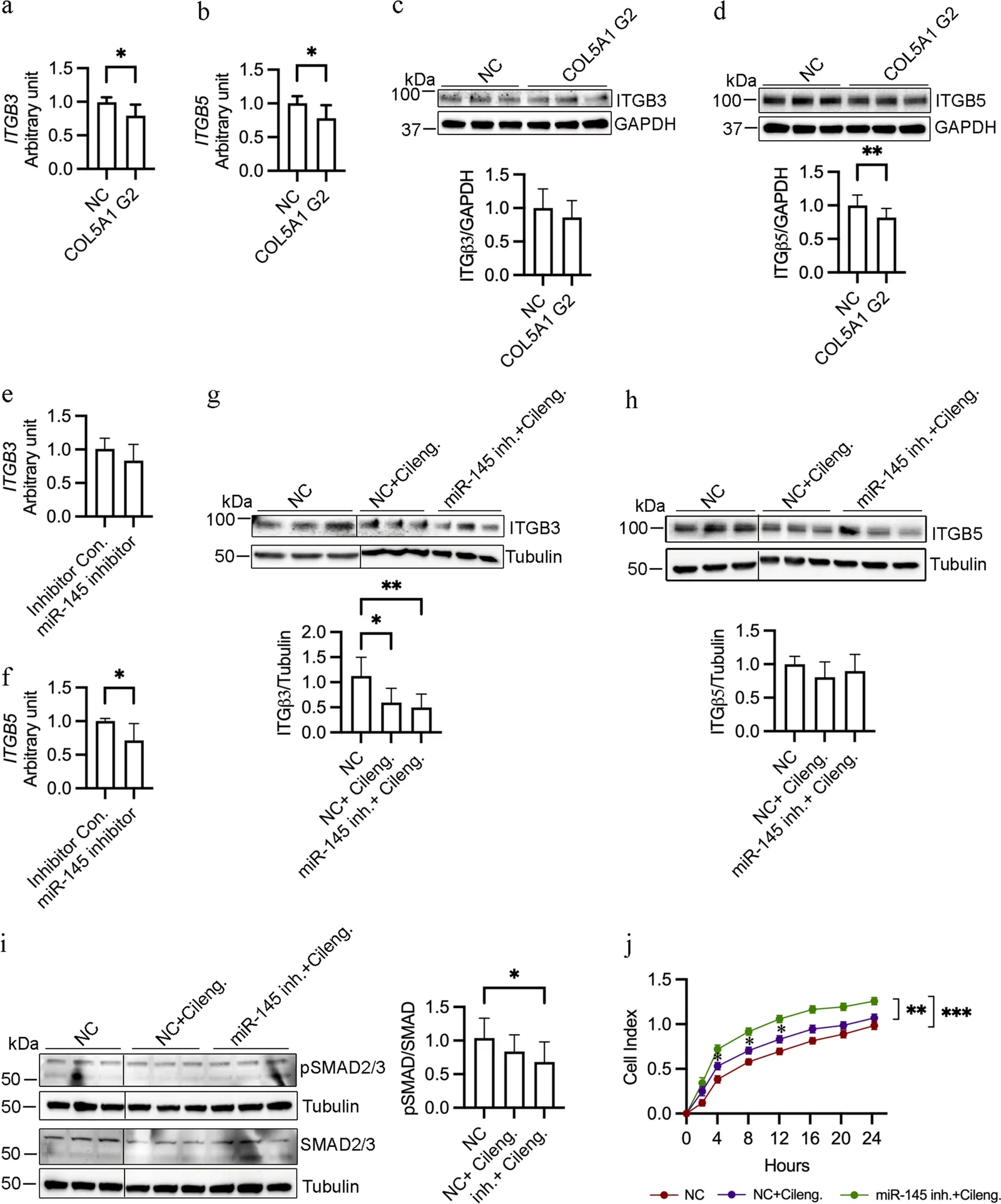
miR-145-5p potentially regulates integrin-mediated pathways independent of COL5A1 in human cardiac fibroblast. **a-b **RT-qPCR analyses of *ITGβ3* (**a**) and *ITGβ5* (**b**) expression in HCF treated with COL5A1 G2, compared to NC. **c **Representative immunoblot and quantification of ITGβ3 in HCF treated with COL5A1 G2, compared to NC. **d **Representative immunoblot and quantification of ITGβ5 in HCF treated with COL5A1 G2, compared to NC. **e-f **RT-qPCR analyses of *ITGβ3* (**e**) and *ITGβ5* (**f**) expressions in HCF treated with miR-145-5p inhibitor compared with inhibitor control. **g-h **Representative immunoblot and quantification of ITGβ3 (**g**) and ITGβ5 (**h**) expressions in HCF either treated with cilengitide alone or in combination with miR-145-5p inhibitor, compared to NC. **i **Representative immunoblot and quantification of pSMAD2/3 relative to SMAD2/3 in HCF either treated with cilengitide alone or in combination with miR-145-5p inhibitor, compared to NC. The expressions of pSMAD2/3 and SMAD2/3 were normalized to tubulin in the respective gels, then the phosphorylation of SMAD2/3 was quantified by normalizing it to SMAD2/3. **j **Real-time proliferation profiles of HCF treated with cilengitide alone or together with miR-145-5p inhibitor, compared to NC. Data are presented as mean ± SD. Two-sample *t*-test. One-way ANOVA with Tukey adjustment or two-way ANOVA with Dunnett adjustment for multiple comparisons. * *p* < 0.05, ** *p* < 0.01, *** *p* < 0.001

Given that COL5 regulates scar size in an integrin-dependent manner [57], we next investigated whether downregulation of COL5A1 with COL5A1 G2 modulates the expression of integrin alpha V (*ITGαV*), beta 3 (*ITGβ3*), and beta 5 (*ITGβ5*) in HCF. Downregulation of COL5A1 led to significant reduction in the transcript levels of *ITGβ3* and *ITGβ5* (Fig. 8a, b), but had no obvious effect on *ITGαV* (Fig. S4g). Consistent with the transcript level, downregulation of COL5A1 substantially reduced ITGβ5 protein level and induced a trend toward decreased ITGβ3 abundance (Fig. 8c, d). In contrast, the expression levels of lysyl oxidase (*LOX*), a key mediator of collagen cross-linking, and periostin (*POSTN*), an integrin ligand, were unaffected upon downregulation of COL5A1 (Fig. S4h, i). The findings suggest that COL5A1 may not be involved in collagen cross-linking, but could rather play a role in regulating integrin-mediated pathways.

Since miR-145-5p targets COL5A1 and its inhibition results in elevated transcript level of *COL5A1* in HCF, we investigated the expression of *ITGβ5* and *ITGβ3* upon inhibition of miR-145-5p. Unexpectedly, inhibition of miR-145-5p led to a downregulation of *ITGβ5* and a tendency towards decreased *ITGβ3* expression (Fig. 8e, f). To further verify the potential regulatory link between miR-145-5p and integrin-mediated signalling, we treated HCF with cilengitide, an RGD-mimetic integrin inhibitor selective for αvβ3 and αvβ5 [34], either alone or in combination with miR-145-5p inhibitor. In line with a prior study [38], cilengitide induced a downregulation of ITGβ5 in a dose-dependent manner (Fig. S4j). Treatment with 5 µM cilengitide alone reduced ITGβ3 protein level, whereas its combination with miR-145-5p inhibitor intensified the effect (Fig. 8g). In addition, cilengitide alone or in combination with miR-145-5p inhibitor appeared to reduce ITGβ5 (Fig. 8h). A previous study demonstrated that cilengitide significantly inhibits the activation of the TGFβ/Smad pathway [7]. We thus examined the phosphorylation of SMAD2/3 (pSMAD2/3). Cilengitide alone modestly reduced pSMAD2/3 relative to total SMAD2/3, whereas the combined treatment with miR-145-5p inhibitor resulted in a marked reduction (Fig. 8i). Intriguingly, cilengitide accelerated HCF proliferation on fibronectin-coated plates, which was further enhanced by miR-145-5p inhibitor (Fig. 8j). These findings suggest that miR-145-5p may modulate integrin-mediated signalling through mechanisms beyond direct targeting of COL5A1.

## Discussion

In the present study, we observed significant enrichment of DEGs involved in ECM-receptor interaction and ECM organization in 14 dpi zebrafish hearts. In line with our findings, genes involved in ECM organization were also enriched in the intermediate phase of zebrafish heart regeneration during 14–45 dpi [27]. ECM is a dynamic molecular network that provides mechanical support and mediates molecular signalling for maintaining cardiac function [26]. The cardiac ECM undergoes remodelling to form fibrotic scar tissue in response to injury to prevent rupture of the ventricular wall [49]. Adult mammalian hearts exhibit persistent fibroblast activation, sustained COL1/3 secretion, and excessive collagen cross-linking, contributing to a stiff fibrotic scar. In contrast, zebrafish develop transient ECM deposition, activate matrix degradation pathways, and shift the collagen types from stiffer Col1/3 toward types that support tissue plasticity, such as Col4 [42]. This dynamic remodelling enables the efficient breakdown of fibrotic scar tissue and supports cardiomyocyte proliferation. Indeed, we have observed upregulation of *col4a1,* regeneration-associated gene, *mdka*, as well as cardiomyocyte function-related genes, *tpma* and *s100a10b* in 14 dpi zebrafish hearts. In addition, we found that the expression of *col5a1* is enriched in regenerating zebrafish hearts whereas it is decreased in the LV of ICM patients. Similar to our finding, increased transcription levels of col4a2 and col5a1 were previously found in regenerating zebrafish hearts [18].

We found that miR-145-5p was downregulated in 14 dpi zebrafish hearts and upregulated in LV of ICM patients. Similar to our finding, circulating miR-145 level is elevated in AMI patients and positively correlates with infarct size, suggesting that miR-145 can be a biomarker for predicting long-term outcome post AMI [10, 35]. A previous study demonstrated that miR-145 induces fibroblast differentiation to myofibroblasts and potentiates the ability of myofibroblasts to produce mature collagen [54]. Accordingly, downregulated miR-145-5p in 14 dpi zebrafish hearts might contribute to cardiac cell proliferation and reduced fibrosis. Indeed, we demonstrate that inhibition of miR-145-5p reduces COL1 expression and promotes cardiac fibroblasts proliferation and migration. During cardiac regeneration in zebrafish, downregulation of fibroblast ECM production is a key step in the regression of fibrosis [42]. An overall decrease in collagens and ECM stiffness is associated with zebrafish cardiac regeneration [18, 43]. We further illustrated that inhibition of miR-145-5p decreased the expression of ITGβ3 and ITGβ5 and potentiated the suppressive effect of cilengitide on TGF-β/SMAD signalling. This is consistent with a prior study showing upregulation of ITGβ5 and activation of SMAD2/3 signalling in rat cardiac tissue and fibroblasts from spontaneously hypertensive rats, whereas cilengitide attenuated TGF-β1 release and reduced the expression of fibrotic proteins, including COL1, in primary rat cardiac fibroblasts [38]. Fibronectin, an essential extracellular matrix glycoprotein, binds αv-class integrins through the RGD motif [4] and regulates cell migration and proliferation [44]. Notably, fibronectin lacking the integrin-binding RGD motif promotes cell growth more strongly than wild-type fibronectin [44]. In agreement with this, cilengitide-induced blockage of αvβ5 and αvβ3 stimulated HCF proliferation on fibronectin-coated plates, and miR-145-5p inhibition further enhanced this response in the current study. Collectively, downregulated miR-145-5p in cryoinjured zebrafish hearts at 14 dpi may promote fibroblast proliferation and migration potentially through modulating integrin-mediated pathways.

We further demonstrated that *COL5A1* is a target of miR-145-5p. Thus, the effect of miR-145-5p on suppression of fibroblast activation, proliferation, and migration could be mediated also via targeting *COL5A1*. In fact, downregulation of COL5A1 suppressed HCF proliferation and migration. Studies have shown that COL5 deficiency leads to cardiac fibroblast activation and develops a large fibrotic scar area with disrupted architecture in mice [57]. Consistently, we found downregulation of col5a1 attenuated the fibrotic scar resolution in zebrafish, further confirming the role of col5a1 in regulating cardiac scar remodelling. However, downregulation of COL5A1 decreased the expression level of COL1A1 and had no significant effect on αSMA in HCF, suggesting that COL5A1 mainly modulates collagen matrix organization rather than directly driving myofibroblast differentiation. COL5 is key regulator of collagen fibrillogenesis and matrix organization [55, 57], particularly in the assembly of COL1 into mature and functional fibrils [46]. A prior study has shown that COL5 expression spatially overlaps with either COL3 or COL1 in the injury region [57]. Our Cytoscape analysis revealed a core cluster formed by COL5A1-3 in conjunction with other collagen genes, such as COL1A1/2 and COL3A1. This indicates a pivotal role of COL5A1 in the collagen network, particularly in cardiac fibroblasts where COL5A1/2 is strongly co-expressed with COL1A1/2 and COL3A1. In the cornea, COL5 co-assembles with COL1 to form heterotypic fibrils, and the proportion of COL5 directly affects fibril diameter, with higher levels resulting in smaller and more uniform fibrils [5]. Whether downregulation of COL5A1 disrupts heterotypic fibril formation and leads to a feedback decrease in COL1A1 expression in the present study requires further investigation. We further illustrated that downregulation of COL5A1 decreased the expression of ITGβ3 and ITGβ5. Interestingly, a significantly greater fraction of Col5a1-deficient mouse cardiac fibroblasts expressed the integrins αvβ3 and αvβ5 [57]. We found no obvious changes in the expression of *LOX* and *POSTN* in fibroblasts upon downregulation of *COL5A1*. In contrast, Col5a1 deficient cardiac fibroblasts exhibit a significant upregulation of *LOX* and *POSTN* [57]. This discrepancy may be attributed to differences in experimental set-ups, tissue- vs. fibroblast-specific responses, and detection methods, transcriptomic profiling vs. qRT-PCR. Taken together, miR-145-5p regulates fibroblast behaviour partially via COL5A1.

We demonstrated that DEGs associated with inflammatory response are significantly upregulated in 14 dpi zebrafish hearts. Consistent with our findings, DEGs involved in immune response are highly enriched during the early response to cryoinjury in zebrafish hearts [27]. Acute inflammatory response to cardiomyocyte necrosis is fundamental for cardiac healing [14], but unresolved inflammation can become chronic and cause adverse LV remodelling and dysfunction [1]. Distinct macrophage populations derived from neonatal and adult lineages are important determinants of tissue repair and inflammation, respectively [2, 29]. Macrophages from neonatal lineage provide necessary signals to drive cardiac repair via cardiomyocyte proliferation and angiogenesis following injury, whereas in adults these are replaced by monocyte-derived macrophages that are proinflammatory and lack reparative activities after infarction [2, 29]. Pro-inflammatory cytokines*,* such as *IL6*, *IL1β*, and *TNFα,* are markedly increased in human ICM hearts [47], whereas no substantial increase is detected in zebrafish hearts at 14 dpi in the present study. This suggests that the zebrafish heart regenerative process may involve unique immune regulatory mechanisms that suppress a sustained inflammatory response.

Our data indicate that DEGs involved in oxidative phosphorylation and fatty acid metabolism are downregulated in 14 dpi zebrafish hearts. Metabolic reprogramming from mitochondrial oxidative phosphorylation to glycolysis is a critical mechanism driving cardiomyocyte proliferation and regeneration in zebrafish [16, 23]. A switch from anaerobic glycolysis in neonatal cardiomyocytes to mitochondrial aerobic respiration in mature cardiomyocytes results in reactive oxygen species generation, which in turn triggers a DNA damage response and induces cell-cycle arrest in mammals [31, 40]. This suggests that stimulation of a fetal-like metabolic state and reduction of mitochondrial-dependent oxidative stress could be an important strategy to trigger cardiomyocyte proliferation and cardiac regeneration.

In conclusion, we found activation of the molecular pathways involved in ECM organization and inflammatory response and downregulation of genes associated with mitochondrial organization and oxidative phosphorylation in zebrafish cryoinjured hearts at 14 dpi. Furthermore, we demonstrated that COL5A1 is a target of miR-145-5p and their expression displays opposite patterns in the regenerating zebrafish heart versus the decompensated human heart. Downregulation of col5a1 led to increased collagen accumulation and impaired fibrotic scar resolution in zebrafish cryoinjured hearts. We further illustrated that miR-145-5p modulates collagen expression and cardiac fibroblasts behavior partially via targeting COL5A1 and through COL5A1-independent integrin-mediated pathways. We thus envision that the miR-145-5p-collagen V-integrin axis could be employed as a putative target for therapies promoting cardiac regeneration after MI.

## Supplementary Information

Below is the link to the electronic supplementary material.

Supplementary file1 (TIF 372 KB)

Supplementary file2 (TIF 555 KB)

Supplementary file3 (TIF 476 KB)

Supplementary file4 (TIF 639 KB)

Supplementary file5 (DOCX 16 KB)

Supplementary file6 (XLSX 17491 KB)

Supplementary file7 (XLSX 98 KB)

Supplementary file8 (XLSX 26 KB)

## Data Availability

The RNA-seq dataset will be deposited in Gene Expression Omnibus.
