# Supplementary material for: MiR-145-5p modulates collagen production and fibroblast behaviour after cardiac injury partially via COL5A1
Source: Basic Res Cardiol. 2026 Aug 3;121(5):965–86. doi: 10.1007/s00395-026-01199-0 (PMC13593628; doi:10.1007/s00395-026-01199-0)
Supplement: Supplementary file 1 [file 395_2026_1199_MOESM5_ESM.docx]

**MiR-145-5p modulates collagen production and fibroblast behaviour after cardiac injury partially via COL5A1**

**Wang *et al.***

**Supplementary Figure Legends**

**Supplementary Fig. 1** The top two enriched pathways in zebrafish cryoinjured hearts at 14 dpi visualized using Pathview.

Significantly enriched DEGs are involved in ECM-receptors interaction (left panel) and leukocyte trans-endothelial migration (right panel) in cryoinjured hearts. Upregulated genes are marked in red, downregulated in green.

**Supplementary Fig. 2** The top two downregulated pathways in zebrafish cryoinjured hearts at 14 dpi visualized using Pathview.

Significantly downregulated DEGs are involved in mitochondrial oxidative phosphorylation (upper panel) and fatty acid degradation (lower panel) in cryoinjured hearts. Downregulated genes are marked in green, upregulated in red.

**Supplementary Fig. 3** **a-b** RT-qPCR analyses of the expression of *KLF5* (a) and *AIFM1* (b) in HCF when overexpressing miR-145-5p. **c-d** RT-qPCR analyses of the expression of *KLF5* (c) and *AIFM1* (d) in HCF when inhibiting miR-145-5p. **e** Cytoscape analysis of collagen (COL) network. COL5A1-3 form a core cluster with COL1A1/2, COL2A1, COL3A1 and COL11A1/2. *KLF, Krueppel-like factor 5; AIFK1, Apoptosis-inducing factor mitochondria associated 1.*

Data are presented as mean ± SD. Two-sample *t*-test. * *p* < 0.05, ** *p* < 0.01.

**Supplementary Fig. 4** **a** RT-qPCR analysis of *COL5A1* expression in HCF treated with COL5A1 GapmeR DR1 (COL5A1 G1) or DR2 (COL5A1 G2), compared to negative control (NC). **b** Representative immunoblot and quantification of COL5A1 in HCF treated with either COL5A1 G1 or G2, compared to NC. **c** RT-qPCR analysis of *ACTA2* expression in HCF either treated with COL5A1 G2 alone or together with miR-145-5p inhibitor, compared to NC. **d** Representative immunoblot and quantification of αSMA in HCF either treated with COL5A1 G2 alone or together with miR-145-5p inhibitor, compared to NC. **e-f** RT-qPCR analyses of the expression of *col5a1* in livers (e) and kidneys (f) of zebrafish injected with COL5A1 G2 compared with those injected with NC.

RNA was extracted from 1-2 pooled livers or kidneys. The number of RNA samples used for RT-qPCR analyses: NC *n* = 3; COL5A1 G2 *n* = 3. g-i RT-qPCR analyses of the expression of *ITGAV* (g), *LOX* (h), and *POSTN* (i) in HCF treated with COL5A1 G2 or NC. *ACTA2,* *Alpha smooth muscle actin; ITGAV, Integrin alpha-V; LOX, Lysyl oxidase; POSTN, Periostin.* J Representative immunoblot and quantification of ITGβ5 in HCF treated with cilengitide at 1, 5, and 10 µM, compared to control.
